# Native extracellular matrix preserves mesenchymal stem cell “stemness” and differentiation potential under serum-free culture conditions

**DOI:** 10.1186/s13287-015-0235-6

**Published:** 2015-12-01

**Authors:** Rubie Rakian, Travis J. Block, Shannan M. Johnson, Milos Marinkovic, Junjie Wu, Qiuxia Dai, David D. Dean, Xiao-Dong Chen

**Affiliations:** Department of Comprehensive Dentistry, University of Texas Health Science Center at San Antonio, 7703 Floyd Curl Drive, San Antonio, TX 78229-3900 USA; Periodontics Graduate Program, Wilford Hall 59th Medical Wing, 2133 Pepperrell Street, Building 3352, Joint Base San Antonio, Lackland, TX 78236 USA; Department of Orthodontics, Fourth Military Medical University, School of Stomatology, 145 West Chang-le Road, Xi’an, Shaanxi Province 710032 P.R. China; Research Service, Audie Murphy VA Medical Center, South Texas Veterans Health Care System, 7400 Merton Minter Boulevard, San Antonio, TX 78229-4404 USA

**Keywords:** Mesenchymal stem cells, Extracellular matrix, Serum-free media, Stem cell expansion

## Abstract

**Introduction:**

Bone marrow-derived mesenchymal stem cells (BM-MSCs) for clinical use should not be grown in media containing fetal bovine serum (FBS), because of serum-related concerns over biosafety and batch-to-batch variability. Previously, we described the preparation and use of a cell-free native extracellular matrix (ECM) made by bone marrow cells (BM-ECM) which preserves stem cell properties and enhances proliferation. Here, we compare colony-forming ability and differentiation of MSCs cultured on BM-ECM with a commercially available matrix (CELLstart™) and tissue culture plastic (TCP) under serum-free conditions.

**Methods:**

Primary MSCs from freshly isolated bone marrow-derived mononuclear cells or passaged MSCs (P1) were grown in serum-containing (SCM) or serum-free (SFM) media on BM-ECM, CELLstart™, or TCP substrates. Proliferation, cell composition (phenotype), colony-forming unit replication, and bone morphogenetic protein-2 (BMP-2) responsiveness were compared among cells maintained on the three substrates.

**Results:**

Proliferation of primary BM-MSCs was significantly higher in SCM than SFM, irrespectively of culture substrate, suggesting that the expansion of these cells requires SCM. In contrast, passaged cells cultured on BM-ECM or CELLstart™ in SFM proliferated to nearly the same extent as cells in SCM. However, morphologically, those on BM-ECM were smaller and more aligned, slender, and long.

Cells grown for 7 days on BM-ECM in SFM were 20–40 % more positive for MSC surface markers than cells cultured on CELLstart™. Cells cultured on TCP contained the smallest number of cells positive for MSC markers. MSC colony-forming ability in SFM, as measured by CFU-fibroblasts, was increased 10-, 9-, and 2-fold when P1 cells were cultured on BM-ECM, CELLstart™, and TCP, respectively. Significantly, CFU-adipocyte and -osteoblast replication of cells grown on BM-ECM was dramatically increased over those on CELLstart™ (2X) and TCP (4-7X). BM-MSCs, cultured in SFM and treated with BMP-2, retained their differentiation capacity better on BM-ECM than on either of the other two substrates.

**Conclusions:**

Our findings indicate that BM-ECM provides a unique microenvironment that supports the colony-forming ability of MSCs in SFM and preserves their stem cell properties. The establishment of a robust culture system, combining native tissue-specific ECM and SFM, provides an avenue for preparing significant numbers of potent MSCs for cell-based therapies in patients.

## Introduction

Bone marrow-derived mesenchymal stem cells (BM-MSCs) not only replicate to produce identical daughter stem cells (self-renewal) but can differentiate into many distinct cell types, including osteoblasts, adipocytes, chondrocytes, and myocytes [1–4]. Throughout life, MSCs are continually involved in tissue regeneration and may be potentially useful as cell-based therapies for a number of diseases such as graft-versus-host disease, myocardial infarction, and diabetes (http://clinicaltrials.gov/). However, because of their relative scarcity in adult bone marrow (approximately 0.001 %) [5, 6], MSCs must be expanded *in vitro* to obtain sufficient numbers for basic research studies or clinical applications. Typically, the growth of MSCs requires a medium containing 10 % to 15 % fetal bovine serum (FBS). For stem cell-based therapies, alternatives to FBS have been sought since there is significant batch-to-batch variation from suppliers. More importantly, there are biosafety concerns, such as xenoimmunization and the risk of disease transmission by known or unknown pathogens (e.g., mycoplasma, viruses, and prions) [7–9].

Efforts by others have focused on developing a defined cell culture system consisting of a three-dimensional (3D) matrix, composed of purified or recombinant matrix proteins, combined with serum-free media (SFM) containing various growth factors for propagating MSCs *in vitro* [10–13]. Although the results using this cell culture system have shown promise when compared with culture on ordinary tissue culture plastic (TCP), these purified or recombinant matrix proteins lack critical components found in bone marrow extracellular matrix (BM-ECM). *In vivo* MSCs are surrounded by a rich ECM, composed of collagens, adhesion proteins, proteoglycans, and growth factors, which forms a unique microenvironment known as the “niche” [14, 15]. In this local microenvironment, MSCs not only receive signals from the ECM but actively remodel it by secreting various matrix components and proteases and depositing storage depots of growth factors. An accurate reconstruction of an authentic BM-ECM from isolated components would be difficult because of its intricate nature.

To preserve stem cell properties during culture, we developed an experimental system which mimics the *in vivo* microenvironment. In our approach, native ECM is systematically produced by mouse or human bone marrow cells and then decellularized [16, 17]. This native ECM is composed of at least 70 different components that include collagens (types I and III), fibronectin, small leucine-rich proteoglycans (biglycan and decorin), and basement membrane constituents (perlecan and laminin). Together, these matrix proteins play key roles in regulating cell adhesion, migration, proliferation, differentiation, and survival [18–21]. Indeed, mouse and human BM-MSCs, cultured on this cell-free BM-ECM, display enhanced attachment and proliferation while retaining their stem cell properties [16, 17]. In addition, we found that BM-MSCs maintained on BM-ECM displayed significantly increased sensitivity to growth factors such as bone morphogenetic protein-2 (BMP-2) [16]. Furthermore, BM-MSCs expanded on BM-ECM *in vitro* and implanted into immunocompromised mice generated five times more bone and eight times more hematopoietic marrow compared with MSCs expanded on plastic. The ability of the ECM to promote retention of MSC properties is due, at least in part, to sequestration of endogenously produced growth factors that control MSC replication and differentiation [16]. Recently, these findings have been independently supported by other groups [22–25].

In the present study, we hypothesize that BM-MSCs, cultured on surfaces coated with BM-ECM, will display significantly improved stem cell properties after expansion compared with cells cultured on TCP or a commercially available matrix (CELLstart™; Gibco Invitrogen, Grand Island, NY, USA), critically evaluated and tested by many research groups, and frequently used for growing human stem cells [10–13, 26], under identical SFM conditions. To test this hypothesis, we examined the capacity of BM-MSCs, after growth in SFM on the various culture substrates, for proliferation, MSC replication, differentiation, and responsiveness to BMP-2.

## Methods

### Preparation of human BM-MSCs

Freshly isolated human bone marrow mononuclear cells, containing BM-MSCs (primary cells) from 20- to 25-year-old donors, were purchased from Lonza Group Ltd. (Walkersville, MD, USA). Viability was more than 98 % via trypan blue exclusion. These primary cells were seeded into TCP plates (Sigma-Aldrich, St. Louis, MO, USA) at 3 × 10^5^ cells/cm^2^ in “expansion medium” containing alpha-minimum essential medium (α-MEM) (Life Technologies, Grand Island, NY, USA), glutamine (2 mM), penicillin (100 U/ml), streptomycin (100 μg/ml; Biofluids, Rockville, MD, USA), and 15 % FBS (Becton Dickinson, Franklin Lakes, NJ, USA) that had been pre-selected for its growth enhancing activity. The cells were cultured for 2–3 weeks, reaching approximately 70–80 % confluence, with half-volume media changes every 3–4 days. Non-adherent cells were removed by washing with phosphate-buffered saline (PBS). The adherent cells (passage 1; P1), identified as MSCs based on high expression (>90 %) of CD73, CD90, and CD105 and no expression of CD45, were detached by using trypsin (0.02 % for 2 minutes at 37 °C), collected by centrifugation, and resuspended in expansion media. At this point, cells were frozen for future use, used in experiments examining cell behavior, or used for the preparation of BM-ECM.

### Preparation of cell-free BM-ECM

BM-ECM was prepared under aseptic conditions by using procedures developed in our lab [16]. Briefly, BM-MSCs (P1-P3 cells) were seeded onto TCP at 6 × 10^3^ cells/cm^2^ and cultured in expansion medium for 15 days with media changes every 3–4 days. During the last 8 days of culture, ascorbic acid (50 μM) (Sigma-Aldrich) was added to the media to stimulate matrix production. At harvest, the BM-ECM was extensively washed with PBS and the cells were removed by treating the matrix with 0.5 % Triton X-100 containing 20 mM NH_4_OH in PBS for 5 minutes at 37 °C. The resulting BM-ECM was washed with PBS an additional three times, followed by extensive washing with sterile distilled water. After the final water wash, the excess fluid was removed, and the ECM was air-dried before storing at 4 °C for up to 4 months. For use in cell culture, the ECM was re-hydrated with PBS for 1 hour at 37 °C, the PBS removed, and culture media added.

### Surface characterization of cell-free BM-ECM and commercial ECM (CELLstart™)

Surface mapping of BM-ECM and CELLstart™ (Gibco Invitrogen) was performed by using a Veeco MultiMode atomic force microscope (Bruker, Santa Barbara, CA, USA) in tapping mode. Arithmetic mean roughness (R_a_) and average maximum height (R_z_) were obtained by measuring 15 samples of each cell culture substrate.

### Proliferation of BM-MSCs maintained on TCP, cell-free BM-ECM, or commercial ECM (CELLstart™) in serum-free media

Both the proliferation of primary bone marrow cells and passaged cells (P1) grown in SFM were examined. For primary cell culture, freshly isolated human bone marrow mononuclear cells from four different 20- to 25-year-old donors (Lonza Group Ltd.) were seeded onto TCP, BM-ECM, or TCP coated with CELLstart™ (Gibco Invitrogen) prepared in accordance with the instructions of the manufacturer, in six-well plates at 5 × 10^5^ cells/cm^2^. Cell cultures were performed in parallel (n = 3) in two different media: SFM, purchased from Gibco Invitrogen, was prepared in accordance with the instructions of the manufacturer, while our regular “expansion medium” (see above) was used for cultures in serum-containing media (SCM). After 3 days in culture, media were removed and replaced by fresh media to remove non-adherent cells. Once MSC colonies appeared, half-volume media changes were performed twice a week for 2 weeks. At harvest, non-adherent cells were removed by washing with PBS. Owing to differences in attachment to the different substrates, two methods of releasing the cells were required to optimize recovery and maintain cell viability (>90 % using trypan blue). Adherent cells on TCP were detached by using trypsin, while cells on CELLstart™ and BM-ECM were detached by using collagenase (type II; 400 U/ml) (Gibco Invitrogen). Irrespectively of the enzyme used, all cells were washed twice with PBS. Cell proliferation was assessed by cell counting by using a hemocytometer and trypan blue staining. Cell viability was consistently more than 90 %.

To examine the growth of passaged cells, early passage (P1) BM-MSCs (6 × 10^3^ cells/cm^2^) were seeded onto TCP alone, BM-ECM, or CELLstart™ in six-well plates as described above for primary cultures. Representative cells were detached by using trypsin (cells on TCP) or collagenase (type II) (cells on ECM-coated plates), and counted after 4, 7, 10, 14, and 21 days of culture. Cell surface markers were examined by using flow cytometry (see “Flow cytometry” section below) to monitor changes in phenotype over time.

In addition, cell morphology was evaluated after 4, 7, and 10 days in culture; at harvest, culture media were removed and the cultures fixed overnight at 4 °C in 1 % paraformaldehyde. The next day the fixative was removed, 1 ml PBS added, and the wells were sealed by using Parafilm (Sigma-Aldrich). Subsequently, cells were viewed and photographed by using phase-contrast microscopy (Olympus IX73; Olympus, Tokyo, Japan). The spreading shape of cells, cultured on the various substrates for 3 days (before they reached confluence), was compared by measuring circularity (in arbitrary units) using Olympus cellSens image analysis software. Circularity (*C*) was calculated as a ratio of cell area (in micrometers squared) to perimeter (in micrometers) by using the formula:

$$ \left(C=\frac{4\pi {A}_{cell}}{p_{cell}^2}\right). $$

Thus, the area of a cell with equal perimeter would have an idealized circular shape and a circularity of “1”. Circularity values are reported as the mean ± standard deviation (SD) of 60 randomly selected cells on a particular culture surface from three independent experiments.

### Flow cytometry

Prior to seeding, baseline confirmation of BM-MSC phenotype was confirmed via fluorescence-activated cell sorting (FACS) analysis. For the present study, SSEA-4, CD73, CD90, CD105, and CD146, known markers of stem cells [27, 28], were analyzed to determine the immunophenotypic profile of the cells before and after culture on the various substrates. Single-cell suspensions (1–2 × 10^6^) were incubated for 30 minutes at 4 °C with 100-μl aliquots (1:10 dilution) of each primary mouse antibody (SSEA-4, CD73, CD90, CD105, and CD146; Santa Cruz Biotechnology, Inc., Santa Cruz, CA, USA). Non-specific isotype IgG was used as a negative control. Antibody-labelled cells were washed twice with staining buffer (PBS containing 5 % fetal calf serum and 0.01 % sodium azide) and then incubated for 20 minutes at 4 °C with 20 μg/ml secondary antibody (FITC-conjugated goat anti-mouse IgG; Santa Cruz Biotechnology, Inc.). Cells were then washed twice with staining buffer and either analyzed immediately or fixed by using 400 μl/tube of 1 % paraformaldehyde in PBS and stored at 4 °C until analyzed. At least 10,000 events per sample were acquired by using a Becton Dickinson FACStar^plus^ flow cytometer to determine the percentage of positively stained cells.

### Colony-forming unit replication assay

MSC colony-forming ability was determined by using a previously described replication assay [17]. Briefly, P1 bone marrow cells were seeded at 6 × 10^3^ cells/cm^2^ onto TCP, BM-ECM, or CELLstart™ and cultured in SFM for 7 days. At harvest, the cells were detached from the various surfaces, counted, and re-seeded onto plastic plates for determination of CFUs (see below). At the time of seeding onto the three different substrates, an aliquot of the P1 cells was also seeded onto plastic plates for determination of the initial numbers of CFUs as described below.

For CFU assay, cells before and after expansion on the three substrates in SFM were plated into six-well plates—100 cells per well for CFU-fibroblasts (CFU-F); 200 cells per well for CFU-adipocytes (CFU-AD) and CFU-osteoblasts (CFU-OB)—in α-MEM containing 15 % FBS. After 14 days in culture, CFU-F colonies were visualized with crystal violet staining. To assess CFU-AD colony formation, the cultures were maintained for an additional 10 days in adipogenic medium (DMEM containing 10 % FBS, 0.5 mM IBMX, 10^−6^ M dexamethasone, 10 μM insulin, and 200 μM indomethacin) [29] and then stained with Oil Red O to visualize the colonies. To assess CFU-OB colony formation, the cultures were maintained for an additional 25 days in osteoblast differentiation medium: expansion medium supplemented with 10^−7^ M dexamethasone (Sigma-Aldrich) and 10^−4^ M L-ascorbate-2-phosphate (Wako Chemicals, Richmond, VA, USA). The CFU-OB colonies were detected by von Kossa staining. The number of CFUs formed, following 7 days of expansion in SFM, was determined as previously described [17]. MSC replication was expressed as the fold change in CFUs during the expansion: total number of CFUs obtained after cell expansion divided by the initial number of CFUs (before the expansion).

### Determination of changes in osteoblast expression following treatment with BMP-2

Early passage (P1) BM-MSCs (6 × 10^3^ cells/cm^2^) were seeded onto TCP, BM-ECM, or CELLstart™ in six-well plates and cultured in SFM as described above for cell proliferation. At day 7, the media were changed to α-MEM containing 2 % pre-selected FBS and the cultures continued for an additional 24 hours. The cells were then treated with recombinant human BMP-2 (60 ng/ml in PBS; Sigma-Aldrich) or vehicle for 48 hours. The dose of rhBMP-2 selected was based on preliminary studies, in which concentrations ranging from 10 to 200 ng/ml were tested and 60 ng/ml was found to provide an optimal response.

Following BMP-2 treatment, total RNA was extracted and reverse-transcribed by using a High Capacity cDNA Archive Kit (Applied Biosystems, Foster City, CA, USA). Amplification of the cDNAs for alkaline phosphatase (ALP), bone sialoprotein (BSP), and Runx2 was performed by real-time PCR by using TaqMan Universal PCR Master Mix and Assay-on-Demand primers (Applied Biosystems). Amplification and detection were accomplished by using an ABI 7500 Real-Time PCR System (Applied Biosystems), and quantification of gene expression was obtained by subtracting the GAPDH threshold cycle (Ct) value from the Ct value of the gene of interest (ALP, BSP, or Runx2). Quantification of transcript expression was expressed as 2^−ΔCt^.

### Statistical interpretation of data

For cell proliferation studies, each data point represented the mean ± standard deviation of three individual cultures. The data were analyzed via multi-way analysis of variance (ANOVA) following confirmation that all assumptions for ANOVA were verified. Post hoc testing was performed by using the Shapiro-Wilks normalcy test. Log values of data points were analyzed, as normalcy was not confirmed for raw data values. *P* values of not more than 0.05 were considered significant. Cells from four different donors (20–25 years old) were obtained from Lonza Group Ltd. and assayed separately. All results were obtained from at least three independent experiments, and each experimental treatment was performed in triplicate.

## Results

**Fig. 1:**
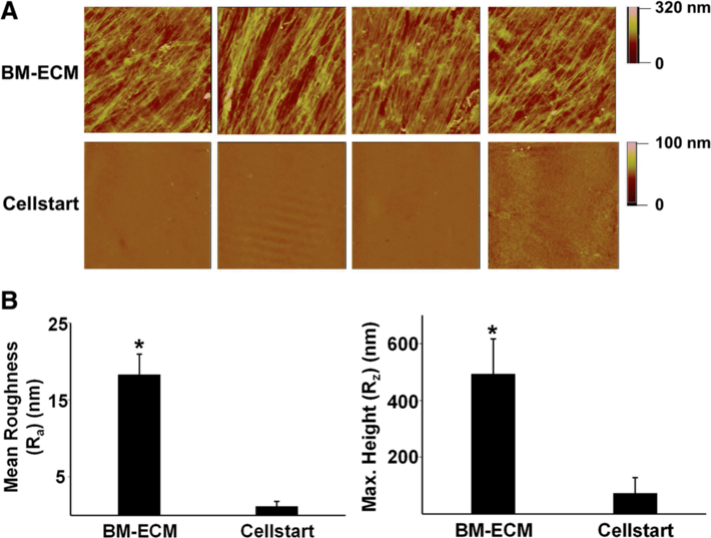
Topographical analysis of BM-ECM versus CELLstart™. **a** Atomic force microscope images highlight the different topographies of the two culture surfaces, BM-ECM versus CELLstart™. The scan areas are 70 × 70 μm for BM-ECM and 20 × 20 μm for CELLstart™. Scale bar: the depth of substrate shown by different colors. **b** Mean roughness and maximal (Max.) height of the two culture surfaces. Mean ± standard deviation was calculated from 15 samples from both cell culture substrates. *P < 0.05, versus CELLstart™. *BM-ECM* bone marrow-derived extracellular matrix

Atomic force microscopy was used to assess the surface characteristics of BM-ECM and CELLstart™. Visible differences in surface topography between the two substrates were easily discerned and quantitatively reflected in significant differences in mean roughness and maximum height (Fig. 1). BM-ECM contained fibers with regional directionality that formed “tracks” and had a mean roughness of approximately 18 nm. In contrast, CELLstart™ was more uniform and smooth and had a mean roughness of approximately 1 nm.

**Fig. 2:**
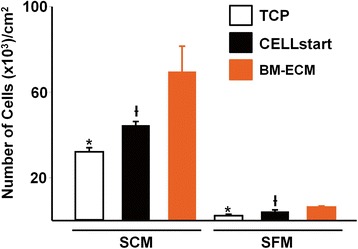
Cell number of primary BM-MSCs after culture in SFM or SCM for 14 days. Primary cultures of BM-MSCs were grown for 14 days on TCP, BM-ECM, or CELLstart™ in SCM or SFM. At harvest, cell number was determined by cell counting after staining with trypan blue. SCM was superior to SFM in promoting cell proliferation, but the overall trend observed with the three culture surfaces remained similar. *P < 0.05, significantly different from BM-ECM and CELLstart™; ^†^ *P* < 0.05, significantly different from BM-ECM. *BM-ECM* bone marrow-derived extracellular matrix, *BM-MSC* bone marrow-derived mesenchymal stem cell, *SCM* serum-containing media, *SFM* serum-free media, *TCP* tissue culture plastic

When freshly isolated primary human bone marrow mononuclear cells were cultured on TCP, BM-ECM, or CELLstart™, the number of cells after 2 weeks was significantly higher in SCM than SFM on all culture surfaces (Fig. 2), suggesting that expansion of primary human bone marrow cells requires SCM. Nevertheless, the primary cells maintained on BM-ECM in SCM or SFM always grew significantly faster than cells maintained on TCP or CELLstart™.

**Fig. 3:**
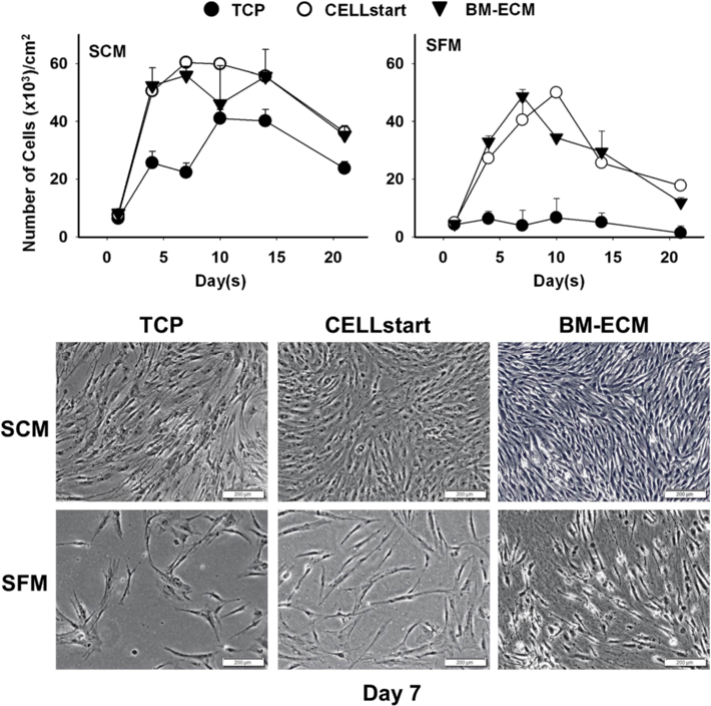
Cell number of early passage BM-MSCs after culture in SFM or SCM for 21 days. Early passage (P1) cultures of BM-MSCs were grown for up to 21 days on TCP, BM-ECM, or CELLstart™ in SCM or SFM. At harvest, cell number was determined by cell counting after staining with trypan blue. Morphology of the cells, after culture on the three different substrates for 7 days in SCM and SFM, is shown in phase-contrast micrographs in the *lower panels*. Scale bar: 200 μm. *BM-ECM* bone marrow-derived extracellular matrix, *BM-MSC* bone marrow-derived mesenchymal stem cell, *SCM* serum-containing media, *SFM* serum-free media, *TCP* tissue culture plastic

Next, we compared the proliferation of early passage (P1) human bone marrow stromal cells on the three culture surfaces in SCM or SFM (Fig. 3). When cells were cultured on either BM-ECM or CELLstart™ in SFM, proliferation followed a temporal pattern similar to that observed with SCM but was less robust. Cells cultured on BM-ECM in SFM reached confluence earlier than those on CELLstart™ (day 7 versus day 10). Except for day 10, where the number of cells on CELLstart™ was significantly higher, the number of cells in SFM cultures was similar on both matrices. In multiple repeat experiments, cells maintained for 7 days on BM-ECM in both SCM and SFM were morphologically smaller and displayed more directional alignment when compared with cells on TCP or CELLstart™ (Fig. 3, lower panels). Cell circularity, after culture for 3 days in SFM, was significantly reduced on BM-ECM versus TCP or CELLstart™ (0.06 ± 0.09, 0.24 ± 0.22, and 0.20 ± 0.23, respectively).

**Fig. 4:**
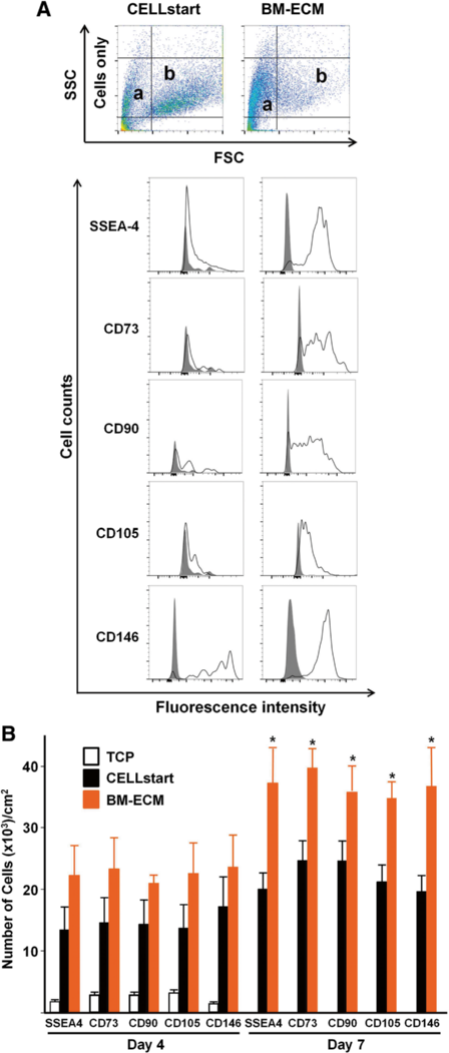
Phenotypic expression of MSC surface markers after culture in SFM for 4 and 7 days. Early passage (P1) cultures of BM-MSCs were grown on TCP, BM-ECM, or CELLstart™ in SFM. Phenotypic expression of MSC-associated markers (SSEA-4, CD73, CD90, CD105, and CD146) was assessed by using flow cytometry. **a** Single-cell suspensions, derived from 7-day cultures on CELLstart™ or BM-ECM, were analyzed by fluorescence-activated cell sorting. In the *top panel*, dot plots of the cell distribution are shown. Relatively smaller cells are found in “range a” (CELLstart: approximately 30 %; BM-ECM: 62 %), whereas relatively larger cells are found in “range b” (CELLstart: approximately 35 %; BM-ECM: 7 %). In the *lower panel*, histograms represent the expression of the indicated markers. Cells were stained with primary non-specific antibody (isotype, IgG) as negative controls (gray peaks). **b** P1 cultures of BM-MSCs were grown on the three culture surfaces for 4 (*left panel*) and 7 (*right panel*) days in SFM. The number of positive cells expressing each marker was determined as a percentage of the total cell population (also see Table 1). Mean ± standard deviation was calculated from three independent experiments. *P < 0.05 versus CELLstart™. *BM-ECM* bone marrow-derived extracellular matrix, *BM-MSC* bone marrow-derived mesenchymal stem cell, *CD*, cluster of differentiation/determinants, *FSC* forward scatter, *MSC* mesenchymal stem cell, *P1* passage 1, *SFM* serum-free media, *SSC* side scatter, *SSEA-4* stage-specific embryonic antigen-4, *TCP* tissue culture plastic

P1 cells that had been cultured on TCP, BM-ECM, and CELLstart™ in SFM for 4 and 7 days were analyzed for cell number and a panel of MSC surface markers (SSEA-4, CD73, CD90, CD105, and CD146). Although cells cultured for 4 days on BM-ECM showed a trend toward greater numbers of MSCs, it never attained statistical significance. In contrast, after 7 days in culture on BM-ECM, the absolute number of cells and the percent positive for MSC markers were significantly increased over cultures on CELLstart™ (Table 1 and Fig. 4b). Dot plots for cell scatter (Fig. 4a) revealed a different cell distribution after culture on CELLstart™ versus BM-ECM (small cells: approximately 30 % versus approximately 62 %; large cells: approximately 35 % versus approximately 7 %, respectively). After both 4 and 7 days, cultures on TCP contained the smallest number of cells and the lowest percent positive for MSC markers by FACS analysis.

**Table 1:** Percent positive cells obtained from cultures on the various substrates

		SSEA-4	CD73	CD90	CD105	CD146
Time in culture	Culture substrate					
4 days	TCP	29 ± 4	45 ± 4	45 ± 5	50 ± 4	24 ± 4
	CELLstart™	46 ± 5	50 ± 7	49 ± 4	47 ± 5	59 ± 7
	BM-ECM	64 ± 6	67 ± 7	67 ± 5	65 ± 6	68 ± 7
7 days	TCP	N/A	N/A	N/A	N/A	N/A
	CELLstart™	45 ± 7	44 ± 8	61 ± 8	46 ± 9	65 ± 8
	BM-ECM	81 ± 10	80 ± 6	81 ± 7	70 ± 6	83 ± 9

*TCP* tissue culture plastic, *BM-ECM* bone marrow-derived extracellular matrix, *N/A* not applicable

Early passage (P1) cultures of BM-MSCs were grown on TCP, BM-ECM, or CELLstart™ in SFM for 4 and 7 days. Phenotypic expression of MSC-associated markers (SSEA-4, CD73, CD90, CD105, and CD146) was assessed by using flow cytometry. The data are the percent positive cells of the total cell population expressing each marker. The experiment was performed in triplicate, and the mean ± standard deviation is shown. Each experiment was performed three times

**Fig. 5:**
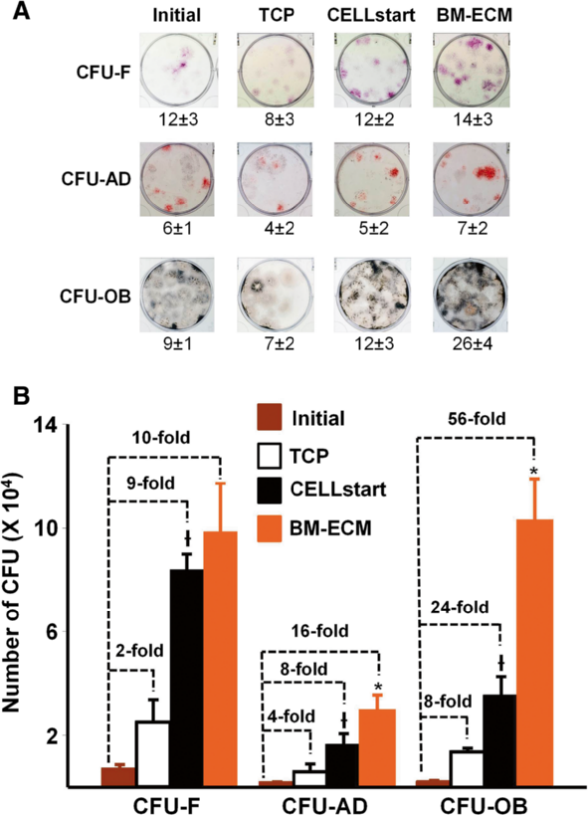
MSC self-renewal and retention of differentiation capacity after culture in SFM on TCP, BM-ECM, and CELLstart™. Early passage (P1) cultures of BM-MSCs were cultured for 7 days on TCP, BM-ECM, or CELLstart™ in SFM. At harvest, the cells were detached, counted, and re-seeded onto plastic plates for CFU assay (CFU-F, CFU-AD, and CFU-OB). Cells, before and after expansion, were plated into six-well plates in α-MEM containing 15 % FBS. After 14 days in culture, CFU-F colonies were stained with crystal violet and counted. Beginning on day 14, cultures for assay of CFU-AD were changed to adipogenic media and cultured for an additional 10 days, and the colonies counted after staining with Oil Red O. Similarly, cultures for assay of CFU-OB were changed to osteogenic media and cultured for an additional 25 days, and the colonies counted after Von Kossa staining. **a** The appearance and frequency of CFU-F, CFU-AD, and CFU-OB assayed at the indicated seeding density before (initial) and after 7 days of expansion on TCP, CELLstart™, and BM-ECM. **b** Over the course of 7 days in culture, MSC replication was represented by the fold changes in the number of colonies after expansion on the various substrates. The mean ± standard deviation was calculated on the basis of three independent experiments. *P < 0.05, versus CELLstart™; and ^Ɨ^ *P* < 0.05, versus TCP and initial. *α-MEM* alpha-minimum essential medium, *BM-ECM* bone marrow-derived extracellular matrix, *BM-MSC* bone marrow-derived mesenchymal stem cell, *CFU* colony-forming unit, *CFU-AD* colony-forming unit-adipocyte, *CFU-F* colony-forming unit-fibroblast, *CFU-OB* colony-forming unit-osteoblast, *FBS* fetal bovine serum, *MSC* mesenchymal stem cell, *P1* passage 1, *SFM* serum-free media, *TCP* tissue culture plastic

MSC colony-forming ability was assessed by measuring the fold increase in CFU-F, CFU-AD, and CFU-OB resulting from MSC replication during 7 days of culture on TCP, BM-ECM, or CELLstart™ in SFM (Fig. 5). P1 cells were divided into aliquots for the determination of CFUs present in the initial P1 population and for expansion on TCP, BM-ECM, and CELLstart™, for the determination of replication as previously described [16]. After 7 days of culture on the various substrates in SFM, the number of cells was obtained. The frequency of CFUs in the cells expanded on the various substrates was determined by plating the same number of cells on TCP for the colony formation assay shown in Fig. 5a. By combining the number of cells obtained from the cultures on the various substrates after 7 days and the frequency of CFUs, we determined that the number of CFU-F was increased 10-, 9-, and 2-fold when P1 cells were cultured on BM-ECM, CELLstart™, and TCP, respectively (Fig. 5b). Furthermore, differentiation capacity of the P1 cells was retained by culture on the various substrates as demonstrated by the CFU-AD and CFU-OB data (Fig. 5b). There was a significant increase in replication, reflected in the increase in CFU-AD and OB, after 7 days of culture on TCP, BM-ECM, or CELLstart™ that was above and beyond that observed for the CFU-F. Specifically, CFU-AD and -OB were increased 16- and 56-fold, respectively, after culture on BM-ECM, which was significantly higher than observed for CELLstart™ (8- and 24-fold, respectively) and TCP (4- and 8-fold, respectively).

**Fig. 6:**
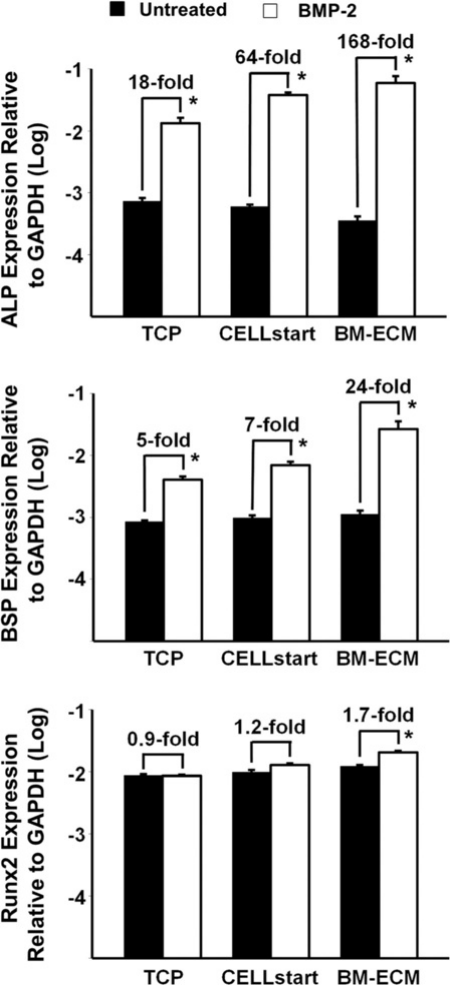
Osteogenic response of BM-MSCs to BMP-2 after culture in SFM on TCP, BM-ECM, and CELLstart™. Early passage (P1) cultures of BM-MSCs were cultured on TCP, BM-ECM, and CELLstart™ in SFM for 7 days; the media were then changed to α-MEM containing 2 % FBS, and the incubation continued for an additional 24 hours before treatment with BMP-2 (60 ng/ml) for 48 hours. Expression of alkaline phosphatase (ALP), bone sialoprotein (BSP), and Runx2 was measured by TaqMan polymerase chain reaction. The mean ± standard deviation was calculated on the basis of three independent experiments. *P < 0.05 versus untreated. *α-MEM* alpha-minimum essential medium, *BM-ECM* bone marrow-derived extracellular matrix, *BM-MSC* bone marrow-derived mesenchymal stem cell, *BMP-2* bone morphogenetic protein-2, *FBS* fetal bovine serum, *GAPDH* glyceraldehyde-3-phosphate dehydrogenase, *P1* passage 1, *Runx2* Runt-related transcription factor-2, *SFM* serum-free media, *TCP* tissue culture plastic

To determine whether MSCs retain their osteoblastogenic response to exogenous BMP-2 after culture on the various substrates in SFM, P1 cells were grown on TCP, BM-ECM, and CELLstart™ for 7 days and then treated with BMP-2 (60 ng/ml) for 48 hours (Fig. 6). The expression of osteoblast markers—alkaline phosphatase (ALP), bone sialoprotein (BSP), and Runx2—was measured by TaqMan PCR. P1 cells cultured on BM-ECM and treated with BMP-2 demonstrate an increase in ALP (168-fold), BSP (24-fold), and Runx2 (1.7-fold) expression compared with untreated controls. In contrast, similarly prepared cells maintained on CELLstart™ or TCP produced a 64- or 18-fold increase in ALP expression, a 7- or 5-fold increase in BSP expression, and a 1.2- or 0.9-fold increase in Runx2 expression, respectively.

## Discussion

Although the composition of CELLstart™ is proprietary, we selected this culture substrate for comparison purposes because (1) CELLstart™ has been well tested for growing human MSCs [10–13, 26], (2) the same company that markets CELLstart™ also sells a compatible serum-free medium, and (3) CELLstart™, which consists of a mixture of human matrix proteins, is an ideal surface to compare with our native ECM because we believe that the native “undetached” ECM retains much of the unique architecture critical for modulating MSC behavior.

In the present study, we compared the topographical characteristics of BM-ECM and CELLstart™ and found their structural organization and surface roughness to be extremely different. One of the major advantages of BM-ECM versus culture surfaces coated with purified ECM proteins is the retention of the ECM’s unique architecture and topographical features that are responsible for cell attachment and migration [30]. In unpublished data, we have observed that MSCs, maintained on BM-ECM, move along the fibers and display directional migration but that MSCs on TCP display random, non-directional behavior. This indicates that culture on authentic ECM minimizes contact inhibition and promotes cell proliferation.

Freshly isolated primary BM-MSCs cultured in SFM proliferated to a much smaller degree than cells grown in SCM, irrespective of the culture surface, during 14 days in culture. In addition, the number of MSCs recovered at the end of culture on BM-ECM was always greater than that after culture on TCP or CELLstart™, irrespective of the culture media (Fig. 2). This was an unexpected finding because an earlier report had demonstrated similar patterns of primary cell growth in SFM versus SCM as long as the cells were maintained on CELLstart™ [11]. In SCM, primary BM-MSCs expanded quickly and the number of cells found after 14 days in culture was related to culture substrate (BM-ECM > CELLstart™ > TCP). Taken together, these results suggest that SFM may not provide sufficient factors to promote MSC proliferation in primary bone marrow mononuclear cell culture. It is possible that other SFMs reported in the literature are superior to the one used in this study. Here, we selected Gibco Invitrogen SFM because it is made by the same supplier as CELLstart™ and has been optimized for use with the CELLstart™ matrix. This allowed us to focus on comparing the behavior of the cells on BM-ECM versus CELLstart™ and minimize differences in various SFM from different suppliers.

The typical constituents found in SCM are numerous and include a wide range of macromolecules such as serum proteins, attachment and spreading factors, growth factors/hormones, and vitamins [31]. However, the key components in SCM required to induce the proliferation of primary MSCs remain to be elucidated and will need to be optimized to complement the growth of MSCs during culture on BM-ECM.

Passaged (P1 and beyond) cells exhibited very similar growth trends in both SCM and SFM when cultured on CELLstart™ or BM-ECM, but not TCP. This suggests that when the cells are grown in SFM they become more dependent on their culture substrate for maintenance of proliferation, and this effect, compared with culture on TCP, is significantly enhanced by culture on 3D matrices. Although passaged cells cultured in SFM on BM-ECM showed a statistically insignificant trend toward increased proliferation compared with CELLstart™, the phenotype of the cells after culture was quite different. Morphologically, cells cultured on BM-ECM (versus CELLstart™) were smaller, spindle-shaped, directionally oriented, and densely packed and appeared more characteristic of viable stem cells. FACS analysis of cells grown on the two 3D matrices further confirmed these morphological observations. Cells cultured on BM-ECM displayed greater expression of MSC markers than cells cultured on CELLstart™, as well as TCP, after 7 days (Fig. 4). These findings indicate that the number of high-quality MSCs was increased when they were maintained on BM-ECM, as compared with TCP or CELLstart™, in a serum-free medium.

To further assess cell quality after expansion on the three different substrates in SFM, MSC colony-forming capacity was measured by using a CFU replication assay (Fig. 5). Consistent with the cell proliferation results, there was no significant difference between cells pre-cultured on BM-ECM versus CELLstart™ in terms of CFU-F replication. However, only a very small proportion of the CFU-F generated cells, pre-cultured on TCP and CELLstart™, were able to differentiate into CFU-OB. In contrast, almost 100 % of the CFU-F, pre-cultured on BM-ECM, formed CFU-OB. The results for CFU-AD were very similar to those observed with CFU-OB. These data suggest that culture on BM-ECM in SFM strongly preserves MSC differentiation capacity when compared with culture on CELLstart™.

Previously, we reported that MSCs maintained on BM-ECM in SCM displayed increased responsiveness to BMP-2 [16]. Since growth factor responsiveness is greatly affected by the local microenvironment of the cells [32], we compared BMP-2 responsiveness of cells maintained on BM-ECM, CELLstart™, and TCP in SFM. Indeed, cells maintained on BM-ECM expressed remarkably higher levels of ALP and BSP in response to BMP-2 when compared with cells grown on CELLstart™ and TCP (Fig. 6). In contrast to ALP and BSP, which are later markers of osteoblast differentiation, Runx2 is a transcription factor involved in early osteoblast differentiation [33]. The relatively low level of Runx2 expression in the present study in response to BMP-2 may be due to the fact that the cells were past the early stage of osteoblast differentiation. These data strongly support the notion that BM-ECM contains a unique architecture and protein composition that specifically modulates the presentation of growth factors to the cells as well as their activation. It remains unclear how the ECM controls cell response to growth factors. However, earlier work has shown that many proteins in the ECM bind various endogenous or exogenous growth factors and serve as a reservoir for more efficiently presenting them to their target cells [34–36].

## Conclusions

In summary, our findings strongly indicate that native BM-ECM provides a unique microenvironment that not only improves the growth of MSCs in SFM but more importantly preserves MSC quality in terms of replication, differentiation, and BMP-2 responsiveness. The establishment of a robust culture system consisting of native tissue-specific ECM and defined SFM will allow us to prepare significant numbers of MSCs, while retaining their stem cell properties, for cell-based therapeutic applications.

## Abbreviations

3D: Three-dimensional
α-MEM: Alpha-minimum essential medium
ALP: Alkaline phosphatase
ANOVA: Analysis of variance
BM: Bone marrow
BM-ECM: Bone marrow-derived extracellular matrix
BM-MSC: Bone marrow-derived mesenchymal stem cell
BMP-2: Bone morphogenetic protein-2
BSP: Bone sialoprotein
CD: Cluster of differentiation/determinants
CFU: Colony-forming unit
CFU-AD: Colony-forming unit-adipocyte
CFU-F: Colony-forming unit-fibroblast
CFU-OB: Colony-forming unit-osteoblast
DMEM: Dulbecco’s modified Eagle’s medium
FACS: Fluorescence-activated cell sorting
FBS: Fetal bovine serum
FITC: Fluorescein isothiocyanate
GAPDH: Glyceraldehyde-3-phosphate dehydrogenase
MSC: Mesenchymal stem cell
P1: Passage 1
PBS: Phosphate-buffered saline
Runx2: Runt-related transcription factor-2
SCM: Serum-containing media
SFM: Serum-free media
SSEA-4: Stage-specific embryonic antigen-4
TCP: Tissue culture plastic
